# Mass Spectrometry-based Lipidomics, Lipid Bioenergetics, and Web Tool for Lipid Profiling and Quantification in Human Cells

**DOI:** 10.21769/BioProtoc.4742

**Published:** 2023-08-20

**Authors:** Liang Cui, Meisam Yousefi, Xin Yap, Clara W.T. Koh, Kwan Sing Leona Tay, Yaw Shin Ooi, Kuan Rong Chan

**Affiliations:** 1 Antimicrobial Resistance Interdisciplinary Research Group, Singapore-MIT Alliance for Research and Technology, Singapore, Singapore; 2 Emerging Infectious Diseases Program, Duke-NUS Medical School, Singapore, Singapore

**Keywords:** Mass spectrometry, Lipid profiling, Lipidomics, Seahorse assay, Bioenergetics, Virus–lipid interactions, Web tool, Clustergram

## Abstract

Lipids can play diverse roles in metabolism, signaling, transport across membranes, regulating body temperature, and inflammation. Some viruses have evolved to exploit lipids in human cells to promote viral entry, fusion, replication, assembly, and energy production through fatty acid beta-oxidation. Hence, studying the virus–lipid interactions provides an opportunity to understand the biological processes involved in the viral life cycle, which can facilitate the development of antivirals. Due to the diversity and complexity of lipids, the assessment of lipid utilization in infected host cells can be challenging. However, the development of mass spectrometry, bioenergetics profiling, and bioinformatics has significantly advanced our knowledge on the study of lipidomics. Herein, we describe the detailed methods for lipid extraction, mass spectrometry, and assessment of fatty acid oxidation on cellular bioenergetics, as well as the bioinformatics approaches for detailed lipid analysis and utilization in host cells. These methods were employed for the investigation of lipid alterations in TMEM41B- and VMP1-deficient cells, where we previously found global dysregulations of the lipidome in these cells. Furthermore, we developed a web app to plot clustermaps or heatmaps for mass spectrometry data that is open source and can be hosted locally or at https://kuanrongchan-lipid-metabolite-analysis-app-k4im47.streamlit.app/. This protocol provides an efficient step-by-step methodology to assess lipid composition and usage in host cells.

**Graphical overview**


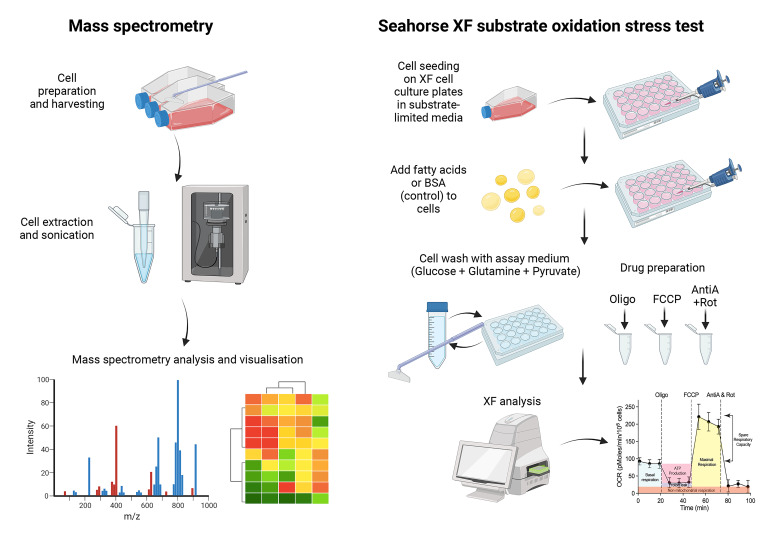


## Background

Lipids are critical for metabolism, signaling, transport across membranes, regulating body temperature, and inflammation. The dysregulation of lipid metabolism or lipid transport can thus disrupt the cell homeostasis and promote oxidative stress that leads to excessive inflammation and cell death. Interestingly, some viruses such as the enveloped viruses have evolved to hijack host cell lipids to augment viral fusion, formation of replication complexes, assembly, and egress. In addition, viruses can also modulate cellular lipid metabolic pathways and leverage intracellular lipid stores to promote viral replication. For instance, dengue virus (DENV) infection can upregulate and re-localize fatty acid synthase (FASN) to the replication complex to increase beta-oxidation for energy production (Heaton et al., 2010; Tang et al., 2014). DENV can also target lipid droplet stores to utilize triglycerides for the production of fatty acids, which are subsequently transported to the mitochondria where they undergo beta-oxidation to synthesize ATP required for viral replication (Zhang et al., 2017). However, much work remains to be done to better understand the virus and lipid interactions responsible for productive virus infection.

RNA interference and CRISPR technologies are useful tools to probe into the lipid metabolic processes required for viral infection. For example, we have recently identified that TMEM41B and VMP1 play a central role in lipid mobilization, mitochondrial beta-oxidation, and global metabolic regulations, to facilitate the replication of flaviviruses and coronaviruses (Yousefi et al., 2022). Deficiency of these proteins resulted in impaired beta-oxidation capacity and dysregulation of the cell lipidome that severely compromised viral infection. To gain insights into the lipid metabolism pathways that are differentially modulated before and after virus infection, we can leverage mass spectrometry to characterize the lipidome. The current protocol describes the detailed procedures required for mass spectrometry in target cells. We have used liquid chromatography–mass spectrometry (LC–MS), the preferred technique for use in metabolomics and lipidomics due to its ability to identify metabolites, even at low concentrations. The method also allows for untargeted lipidomics analysis, so users can simultaneously quantify a variety of lipids in host cells. Furthermore, to evaluate the effects of fatty acid oxidation on cellular bioenergetics, a modified Seahorse assay can be used to ascertain the lipid dependencies for energy production. In combination, these assays provide molecular insights into the role of lipid metabolism in cellular energetics and their potential contribution to virus infection outcome. Finally, we developed a web tool (https://kuanrongchan-lipid-metabolite-analysis-app-k4im47.streamlit.app/) that allows users to plot clustergrams and heatmaps based on the intensity measurements from the LC–MS. While we have used these methods to study virus–lipid interactions in human cells, as lipids are involved in multiple cell processes, we believe that these methods can be more broadly applied to study the role of lipids for other diseases, such as diabetes, obesity, atherosclerosis, and cancer.

In summary, we will describe the detailed protocols for:

1. Sample preparation and cell extraction for LC–MS
2. LC–MS analysis and interpretation
3. Bioinformatics analysis and web tool for data analysis
4. Seahorse bioenergetics analysis for fatty acid oxidation

## Materials and reagents

1. HEK 293FT cells (Invitrogen, catalog number: R70007)
2. Dulbecco’s modified Eagle medium (DMEM) with L-glutamine and sodium pyruvate (Thermo Fisher Scientific, Gibco, catalog number: 11995)
3. 0.22 μm filter (Merck Millipore, catalog number: SLGP0033RS)
4. Seahorse XF RPMI medium (without phenol red, bicarbonate, glucose, pyruvate, or glutamine, contains 1 mM HEPES, adjusted to pH 7.4) (Agilent, catalog number: 103576-100)
5. Seahorse XF calibrant solution [Agilent, 100 mL (catalog number: 03059-000) or 500 mL (catalog number: l100840-000)]
6. L-glutamine (200 mM) (Thermo Fisher Scientific, Gibco, catalog number: 25030081)
7. Fetal bovine serum (FBS) (HyClone, catalog number: SH30396.03)
8. Trypsin-EDTA (0.25%), phenol red (Thermo Fisher Scientific, Gibco, catalog number: 25200072)
9. 10× PBS (Sigma-Aldrich, catalog number: BUF-2040-10X4L)

**Additional materials required for mass spectrometry**

1. Cell scraper (Thermo Fisher Scientific, catalog number: 08-100-241)
2. Tert-butyl methyl ether (MTBE), HPLC Plus (Sigma-Aldrich, catalog number: 650560-1L)
3. Methanol, hypergrade for LC-MS LiChrosolv^®^ (Merck, Supelco, catalog number: 1.06035.2500)
4. Acetonitrile (ACN), hypergrade for LC-MS LiChrosolv^®^ (Merck, Supelco, catalog number: 1.00029.2500)
5. Isopropanol, hypergrade for LC-MS LiChrosolv^®^ (Merck, Supelco, catalog number: 1.02781.2500)
6. Ammonium formate (Honeywell Research Chemicals, Fluka, catalog number: 55674-50g-F)
7. Formic acid, LC/MS grade (Fisher Chemical, catalog number: A117-50)
8. Ultrapure water (H_2_O) (Sartorius Stedim Biotech, arium pro VF)
9. HPLC vial (Agilent, catalog number: 5182-0716)

**Additional materials required for Seahorse assays**

1. Seahorse XFe24 V7 PS cell culture microplate (Agilent, catalog number: 100777-004)
2. Linoleic acid (Sigma-Aldrich, catalog number: L9530-5ML)
3. Oleic acid (Sigma-Aldrich, catalog number: O3008-5ML)
4. Oligomycin A (Sigma-Aldrich, catalog number: 209-437-3)
5. Carbonyl cyanide-4-(trifluoromethoxy) phenylhydrazone (FCCP) (Sigma-Aldrich, catalog number: 206-730-8)
6. Rotenone (Sigma-Aldrich, catalog number: 83-79-4)
7. Antimycin A from *Streptomyces* sp. (Sigma-Aldrich, catalog number: A8674)
8. Substrate-limited media (see Recipes)
9. Seahorse assay media (see Recipes)
10. 10× drugs dilution (see Recipes)

## Equipment

1. Seahorse XFe24 analyzer (Agilent, catalog number: 102238 or S7801A or S7801B)
2. Biological safety cabinet (Esco, BSC Class II)
3. 5% CO_2_, 37 °C incubator
4. CO_2_-free 37 °C incubator
5. Sonicator (Elmasonic S 100 H, model: S100H)
6. Vorterxer (Thermo Scientific, model: M37610-33)
7. Centrifuge (Thermo Fisher Scientific, model: PICO 17)
8. TissueLyzer II (Qiagen)
9. Centrifuges for speed vacuum concentration (Labogene, model: Scan speed 40)
10. Cooling Trap for speed vacuum concentration (ScanLaf A/S, model: Coolsafe 110-4)
11. Mass spectrometry (Agilent Technologies iFunnel QTOF LC-MS, model: G6550B) (Note 2)
12. HPLC system (Agilent 1290 Infinity II, including High Speed Pump, Multisampler, Multicolumn Thermostat)
13. HPLC column, particle size of 1.8 μm, 2.1 mm × 100 mm (Agilent rapid resolution HD Zorbax SB-C18 column, catalog number: 858700-902)

## Software

1. Seahorse Wave software (Agilent, https://www.agilent.com/en/product/cell-analysis/real-time-cell-metabolic-analysis/xf-software/seahorse-wave-desktop-software-740897)
2. Streamlit (Snowflake Inc., https://streamlit.io/)
3. Python (Python Software Foundation, https://www.python.org/)
4. MassHunter Qualitative Analysis 10.0 (Agilent Technologies)
5. MassHunter Profinder 10.0 (Agilent Technologies)
6. Mass Profiler Professional 15.1 (Agilent Technologies)

## Procedure

We describe two protocols: one for the measurement of lipid metabolites in cells and the other to measure lipid utilization for energy production. Most of our experiments have been optimized for HEK 293FT cells, so we recommend users to optimize the cell counts if different cell types are used. Note that users using the mass spectrometry and the Seahorse XF analyzer for the first time should seek training or be accompanied by an experienced user before proceeding.

**Part I: Mass spectrometry protocol**

The protocol for extracting lipid metabolites is as follows:

1. Prepare 10^8^ HEK 293FT cells per replicate for lipid extraction. Users can grow these cells in T75 flasks or in Petri dishes, in DMEM growth medium supplemented with 8% FBS (DMEM GM). We recommend optimizing the cell counts if different cell types are used for measurement.
2. Check the condition of cells before harvesting them for lipid extraction. Ensure that the cells form a confluent monolayer. Floating cells indicate cell death; users should minimize cell death before proceeding with lipid extraction.
3. Pre-chill extraction solvent (made of two parts of methanol mixed with five parts of Milli-Q water) at 4 °C. The solvent is pre-chilled to stop the enzymatic reactions of the cells, so as to preserve the cell states during the harvesting and extraction process.
4. Discard the growth media by either decanting or aspirating with a serological pipette.
5. Wash the cells by adding 5 mL of 1× PBS at room temperature. Discard by decanting or aspirating with a serological pipette.
6. Repeat the wash two times. After the final wash, discard by decanting or aspirating with a serological pipette.
7. Add 560 μL of pre-chilled extraction solvent to the cell culture.
8. Scrape cells with a cell scraper and transfer them into 2 mL Eppendorf tubes.
9. Add 800 μL of pre-chilled MTBE to the tubes (Note 3).
10. Shake the tubes using a TissueLyzer at 30 Hz for 5 min at 4 °C. Repeat two times.
11. Sonicate the tubes in an ultrasonic cleaner pre-filled with ice and water for 15 min. Sonication reduces emulsion formation and assist in the phase separation in liquid–liquid extraction.
12. Centrifuge the tubes at 845× *g* (3,000 rpm) for 15 min at 4 °C.
13. Transfer the upper MTBE layer to a new Eppendorf tube and dry the solvent with a vacuum evaporator at room temperature.
14. Store dried samples in a -80 °C freezer until use.
  The protocol for mass spectrometry analysis is as follows:
15. Reconstitute dried samples in 100 μL of isopropanol/methanol (1:1) (Note 4).
16. Vortex rigorously for 30 s and sonicate for 5 min.
17. Centrifuge at 20,000× *g* (15,000 rpm) for 10 min at 4 °C.
18. Transfer the supernatant into LC vial for LC-QTOF MS analysis.
19. Take 5 μL of each sample for pooling into one vial as the pooled QC sample (Note 5).
20. Prepare mobile phase A water/acetonitrile (60:40) with 10 mM ammonium formate and 0.1% formic acid.
21. Prepare mobile phase B isopropanol/acetonitrile (90:10) with 10 mM ammonium formate and 0.1% formic acid (Castro-Perez et al., 2010).
22. Analyze samples using an Agilent rapid resolution HT Zorbax SB-C18 (2.1 mm × 100 mm, 1.8 μm). The autosampler is set at 4 °C and the injection volume is 5 μL. For users who are not familiar with the protocol or instrument, we recommend doing technical replicates to ensure that the readings obtained are consistent and reproducible.
23. The column temperature is set at 40 °C and the flow rate is set at 0.3 mL/min.
24. The solvent gradient is as follows: 0–2 min, 40% solvent B; 2–12 min, 40%–100% solvent B; 12–15 min, 100% solvent B; 15–15.5 min, 100%–40% solvent B; 15.5–20 min, 40% solvent B.
25. Mass spectrometric analysis is performed in both ESI+ and ESI- modes using the Agilent iFunnel QTOF LC–MS (Figure 1, Note 6). Collect mass data between m/z 100 and 1,000 Dalton at a rate of two scans per second. The major parameter settings are as follows: the ion spray voltage is set at 4,000 V and the heated capillary temperature is maintained at 350 °C. The drying gas and nebulizer nitrogen gas flow rates are 12.0 L/min and 50 psi, respectively. Note that these settings may differ depending on the instrument used, so users will need to optimize their settings for different instruments.
  **Figure 1.**
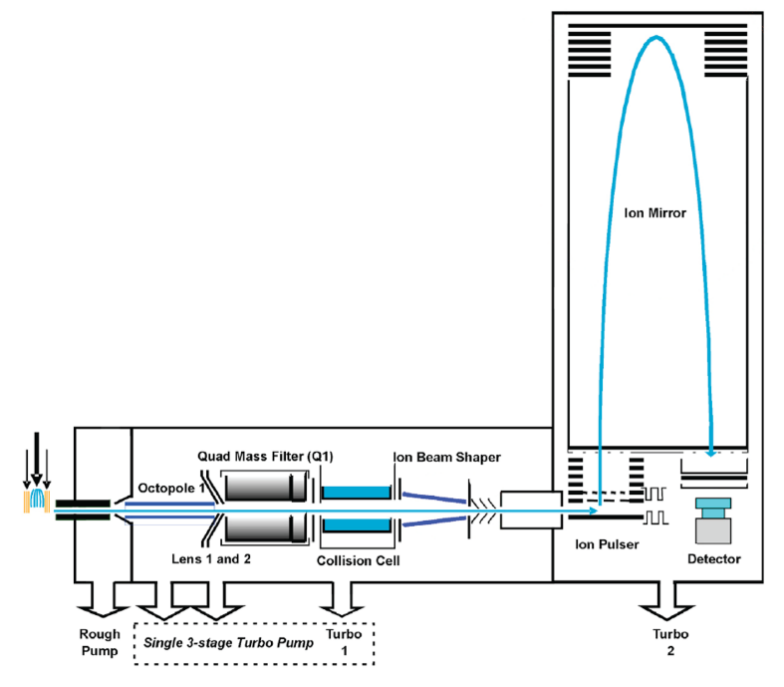
Schematic diagram of Agilent QTOF mass spectrometer. Picture taken and modified from https://www.creative-proteomics.com/support/agilent-6540-uhd-quadrupole-time-of-flight-accurate-mass-mass-spectrometer.htm.

**Part II: Seahorse XF substrate oxidation stress test protocol**

The protocol to evaluate lipid dependencies for energy production is performed over three days. The detailed steps to execute the protocol are as follows:

**Day 1**

**Seeding cells onto Seahorse XFe24 cell culture plate**

1. Dilute HEK 293FT cells to 5 × 10^5^ cells/mL and plate 200 μL of the cell suspension into each well of a SeahorseXFe24 cell culture plate, such that each well has 1 × 10^5^ cells.
2. Incubate at 37 °C with 5% CO_2_ overnight.
**Preparation of substrate-limited media and assay media**
3. Prepare the substrate-limited media supplemented with 1 mM glutamine, 1% FBS, and either 2.5 mg/mL of BSA-conjugated linoleic acid plus 2.5 mg/mL of BSA-conjugated oleic acid or 5 mg/mL of BSA only.
4. Prepare the Seahorse assay media supplemented with either 2.5 mg/mL of BSA-conjugated linoleic acid plus 2.5 mg/mL of BSA-conjugated oleic acid or 5 mg/mL of BSA only.

**Day 2**

**Exchanging cell culture growth media with substrate-limited media**

1. Check the cells under a microscope to ensure that there is a confluent (> 90%) monolayer of cells.
2. Wash the cells in each condition with the respective substrate-limited media (fatty acid–supplemented media or BSA-only media as control) (Figure 2). Remove 150 μL of growth media and add 150 μL of the substrate-limited media. Each condition should be performed in at least three replicates.
3. Repeat the wash a second time by removing 150 μL of media but, this time, adding 200 μL of substrate-limited media to make a final volume of 250 μL of media in each well. Perform the washes gently to ensure that the cells are not dislodged.
4. After the cell washes, check the cells under the microscope to ensure that the cell monolayer is still intact across the wells and that no cells have been dislodged.
5. Leave the plate to incubate at 37 °C with 5% CO_2_ overnight.
  **Figure 2.**
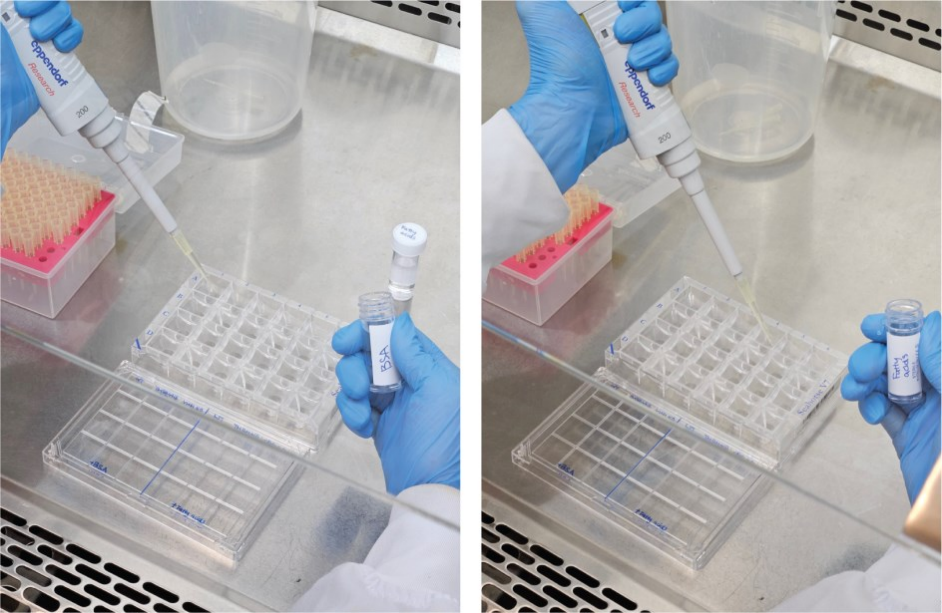
Example of photos showing how BSA or fatty acids can be used for washing and supplemented to the Seahorse XFe24 cell culture microplate to evaluate lipid dependencies for energy production
**Hydrating the sensor cartridges**
6. Hydrate the sensor cartridges according to the manufacturer’s instructions one day prior to running the Seahorse assay.
7. Fill each well of the utility plate with 1 mL of XF calibrant solution.
8. Lower the sensor cartridge carefully into the utility plate and ensure that the sensors are submerged.
9. Leave the plate and cartridge to incubate in a non-CO_2_ 37 °C incubator overnight (Figure 3). Ensure that the incubator is humidified to prevent evaporation of the calibrant.
  **Figure 3.**
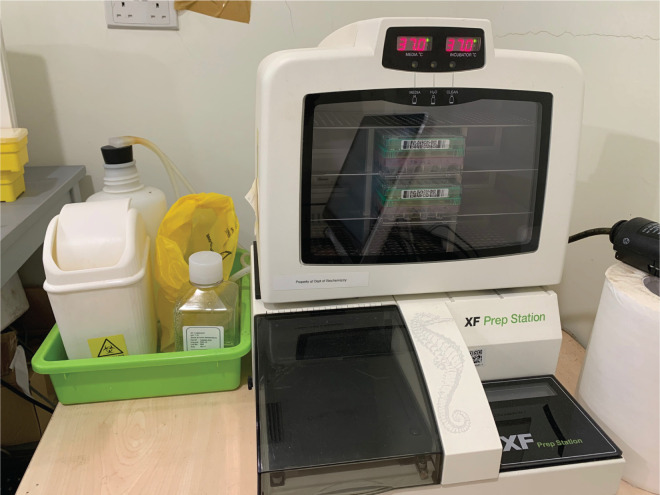
An example of an XF Prep Station that can be used for incubating the plates at 37 °C overnight

**Day 3**

1. Dilute the drugs fresh on the day of assay. Refer to Recipes for details.
2. Wash cells with assay media. Remove 150 μL of media and replace with 150 μL of assay media for each wash. Repeat the wash three times.
3. After the third wash, remove 150 μL of media and add 275 μL of media to top up the volume in each well to 375 μL.
4. Leave the cells to equilibrate in the assay media for at least 30 min in a 5% CO_2_ 37 °C incubator, or in a humidified chamber during transport to a Seahorse facility.
5. Load the drugs into the cartridge (Figure 4) with the following volumes:
  - Port A: oligomycin (70 μL)
  - Port B: FCCP (75 μL)
  - Port C: rotenone + antimycin A (85 μL)
    **Figure 4.**
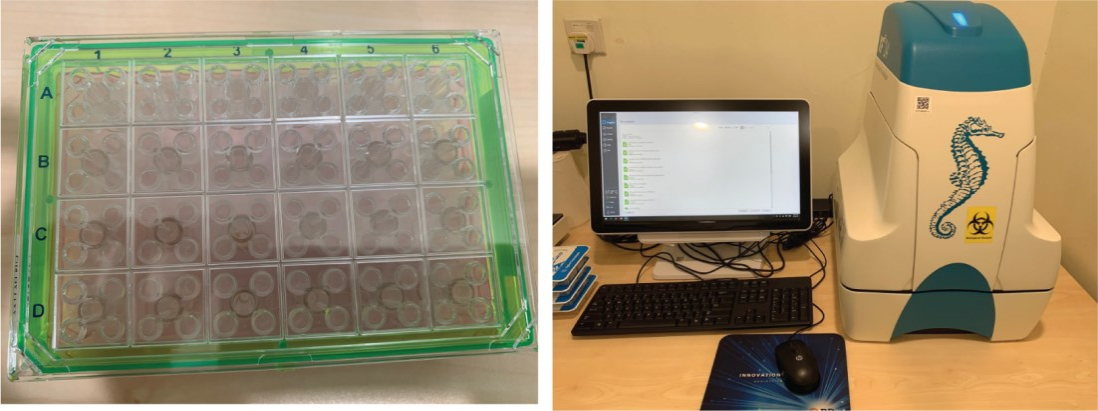
Seahorse XF analyzer. Left: an example of a cartridge for loading the drugs into the Seahorse XF analyzer. By default, each sample is assigned to each quadrant, where Port A can be assigned at the top left, Port B at top right, and Port C at bottom left. Right: the cartridge can then be loaded in a Seahorse XF analyzer.
6. Turn on the Seahorse XF analyzer (Figure 4). Run the Wave software and select the mitochondria stress test assay template, which will assign ports A, B, and C to be oligomycin, FCCP, and rotenone + antimycin A, respectively. Click *Design* to configure the sample positions on the plate map. A standard Seahorse protocol is set up as such:
  - Calibrate.
  - Equilibrate.
  - Baseline readings: loop three times of mix (3 min), wait (2 min), and measure (3 min).
  - Inject port A: loop three times of mix (3 min), wait (2 min), and measure (3 min).
  - Inject port B: loop three times of mix (3 min), wait (2 min), and measure (3 min).
  - Inject port C: loop three times of mix (3 min), wait (2 min), and measure (3 min).
  - End program.
7. Load the sensor cartridge with calibrant plate onto the machine tray and start the calibration step.
8. Once the calibration is done, replace the calibrant plate with the cell culture plate and continue the run with the pre-set protocol.

## Data analysis

**Figure 5.**
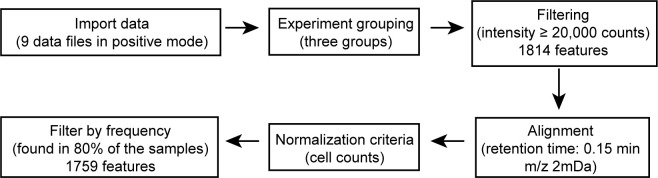
Data processing pipeline of mass spectrometry data in positive mode using Agilent MassHunter Profinder and Mass Profiler Professional software

For molecular feature (a mass spectrometry signal that represents a chemical compound) extraction, raw spectrometric data can be analyzed by MassHunter Profinder and Mass Profiler Professional software (Figure 5). The molecular features are characterized by retention time (RT), chromatographic peak intensity, and accurate mass, which can be obtained by using the Molecular Feature Extractor algorithm. A tolerance window of 0.15 min and 2 mDa was used for alignment of RT and *m/z* values of the features, and only features with an intensity ≥ 20,000 counts and found in at least 80% of data files in least one sample group were kept for further processing. Lipid identities were assigned based on the accurate mass measurement (mass error within ± 5 ppm) and MS/MS fragmentation patterns. Specific standards can be added to confirm the identities of the lipids.

**Metabolites differential analysis**

Intensity values of the different metabolites from LC–MS can be visualized on heatmaps to determine their expression values between the different conditions. Users can consider performing a Z-score transformation to normalize the expression values between the different conditions for each metabolite, allowing for direct comparisons to be made across the different metabolites. Alternatively, users can normalize the expression values as log2-fold changes compared to controls. These calculations can be performed using Microsoft Excel or by using established library packages found in Python and R.

**Figure 6.**
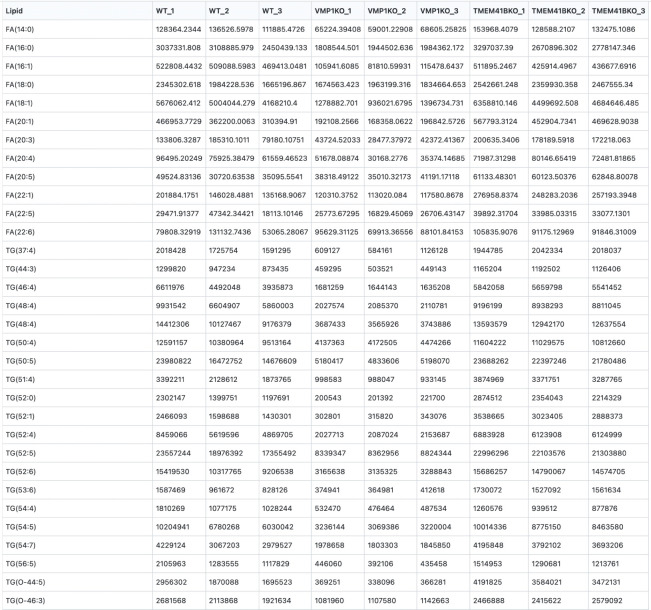
Raw data format for heatmap analysis in the web tool. File containing raw intensity values for the respective conditions from the mass spectrometry can be saved in a .xlsx or .csv format, to be subsequently used in the web tool to plot heatmap and clustermaps. Users can download this demo dataset at https://github.com/kuanrongchan/lipid_metabolite_analysis/blob/main/Lipids_VMP1_TMEM41B_KO.csv.

**Figure 7.**
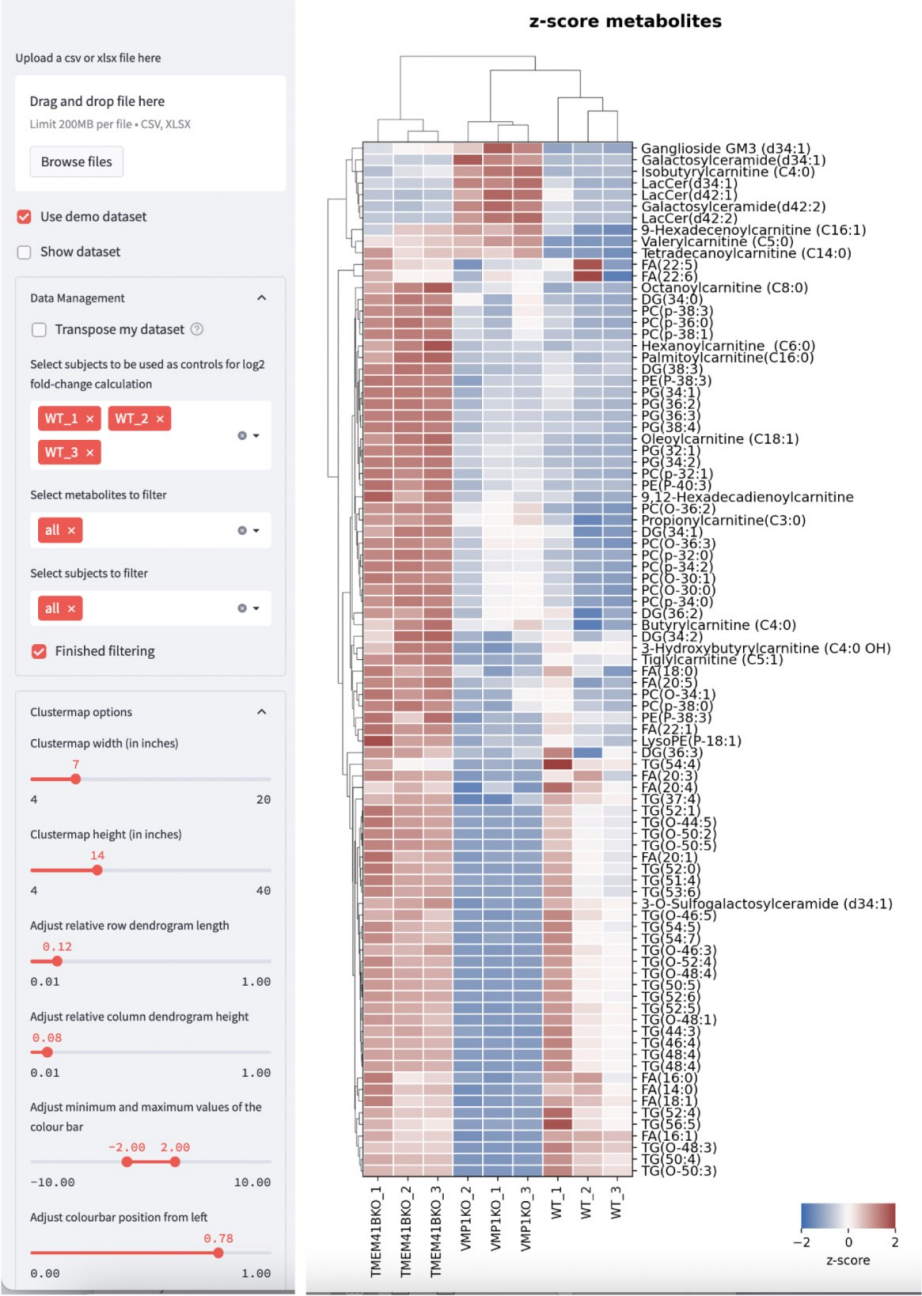
Web tool interface that renders clustergrams based on Z-score or log2-fold change values. Users can customize the settings on the left side bar and click on the *Finished filtering* checkbox to render the clustergram. Dataset based on Yousefi et al. (2022). Clustergram based on Z-score is shown.

To facilitate data visualization, we have also created a web tool where users can directly upload their Excel files with expression values obtained from mass spectrometry analysis to plot clustergrams and heatmaps. The web tool can be accessed at https://kuanrongchan-lipid-metabolite-analysis-app-k4im47.streamlit.app/. To use the web tool, the first column should contain the identifiers (in this case the lipid metabolites) and the subsequent columns can be filled with expression or intensity values (Figure 6). Alternatively, users can use the demo dataset published by Yousefi et al. (2022) at https://github.com/kuanrongchan/lipid_metabolite_analysis/blob/main/Lipids_VMP1_TMEM41B_KO.csv to understand how the data file can be prepared, and to familiarize themselves with the features of the web tool. After uploading the data file, users can then use the sidebar within the web tool to assign the column(s) that contain the control samples (Figure 7). If replicates of the controls are assigned, the mean value will be used for the fold-change (log2-transformed) (log2FC) calculations. This assignment of the control group will allow the web tool to plot the log2-transformed fold-difference values in the treatment conditions as compared to the control conditions. The web tool can also plot relative values based on a filtered list of metabolites, and users can customize the dimensions of the clustergram on the side bar (Figure 2). Finally, after adjusting the settings, users can click on the *finished filtering* checkbox, and the clustergrams for Z-scores and log2FC values will be rendered (Figure 7). The codes for the web tool are also provided publicly at the GitHub repository at: https://github.com/kuanrongchan/lipid_metabolite_analysis/blob/main/app.py.

**Seahorse XF substrate oxidation stress test analysis**

**Figure 8.**
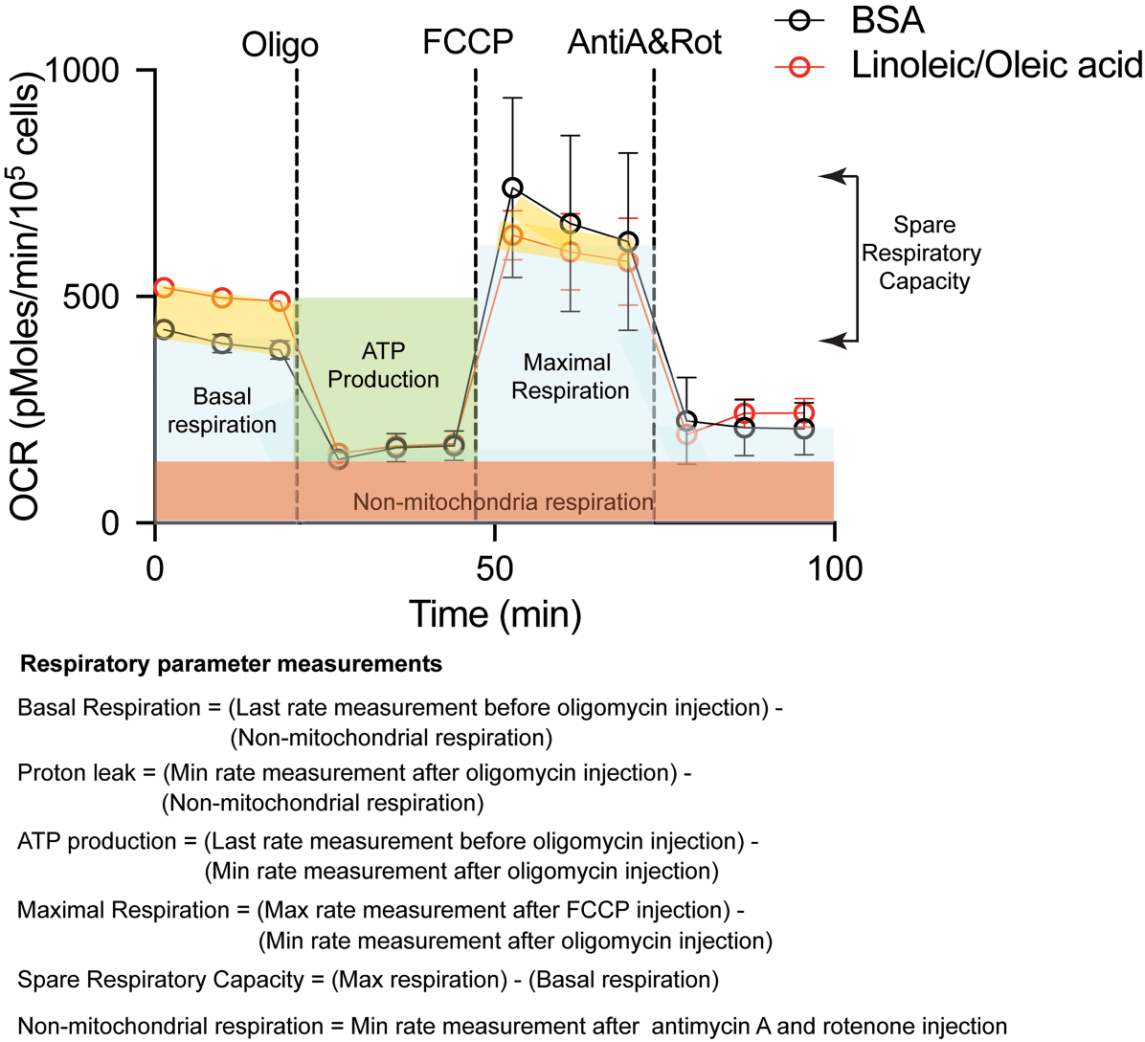
Measurements of the different respiratory parameters in presence of BSA or with supplementation of fatty acids (Linoleic/Oleic acid) in HEK 293FT cells

The mitochondria serve as an important organelle for energy production through oxidative phosphorylation. The mitochondrial respiration test measures the amount of oxygen consumed by the cell, and the supplementation of lipids will determine if more energy is produced via fatty acid oxidation. In addition, various inhibitors of the mitochondria respiratory chain are added to evaluate the contribution of the different mitochondrial complexes in cell respiration. The key respiratory parameters that can be measured are: (i) basal respiration, which is the initial oxygen consumption rate (OCR) minus the non-mitochondrial respiration, (ii) ATP-linked OCR, which is determined after the addition of oligomycin that inhibits ATP synthase, (iii) proton leak, which is the difference between ATP-linked OCR and non-mitochondrial respiration, (iv) maximal respiration, which is induced after addition of carbonyl cyanide-4-(trifluoromethoxy)phenylhydrazone (FCCP), a potent mitochondrial oxidative phosphorylation uncoupler, (v) spare respiratory capacity, which is the difference between maximal respiration and basal respiration, and (vi) non-mitochondrial respiration, which is the OCR after addition of rotenone (complex I inhibitor) and antimycin A (complex III inhibitor). The difference in measurements between these parameters in lipid-supplemented media compared to BSA-supplemented media will indicate the contribution of the fatty acids to mitochondrial respiration (Figure 8).

**Figure 9.**
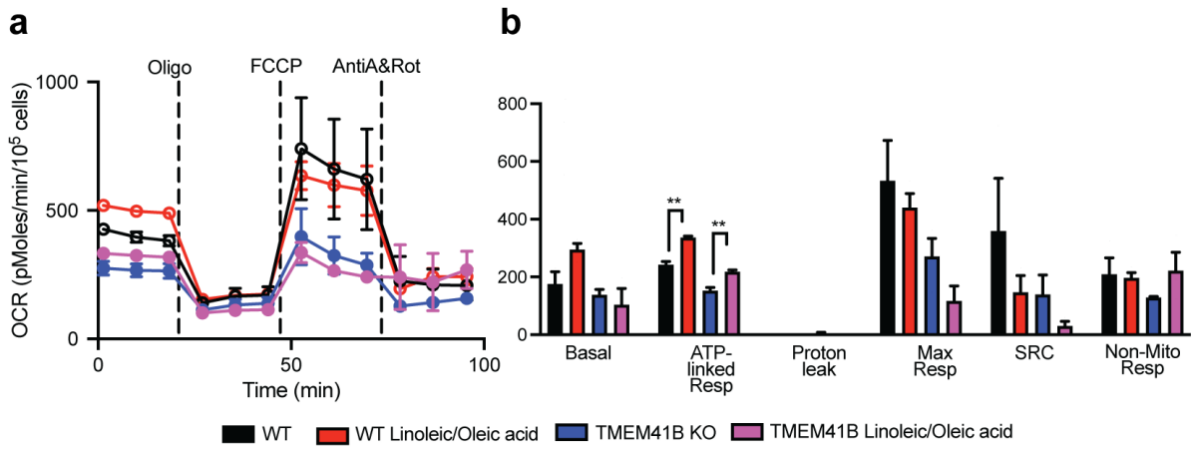
Measurements of the different respiratory parameters in presence of BSA or with supplementation of fatty acids (linoleic/oleic acid) in HEK-293 wild-type (WT) or TMEM41B knock-out (TMEM41B KO) cells

In our illustrated example, we used HEK 293FT cells and evaluated the differences in fatty acid oxidation in wild-type cells compared to CRISPR-knockout cells (e.g., TMEM41B knock-out) (Figure 9). The supplementation of linoleic and oleic acid to wild-type cells increases ATP production through ATP-linked respiration. Similarly, TMEM41B-deficient cells promoted ATP production, although the maximal respiration and the spare respiratory capacity were not significantly altered with the addition of linoleic and oleic acid (Figure 9b). More details of the mechanisms of TMEM41B and VMP-1 in facilitating dengue virus infection in HEK 293FT cells can be found in the publication by Yousefi et al. (2022).

## Notes

1. We recommend performing each experiment in at least triplicates to demonstrate the reproducibility and consistency of the data. Technical replicates should be considered if users are inexperienced with the protocol or instruments. Note that the experiments have been optimized in HEK 293FT cells, so if different cells are used, users will need to optimize the cell counts. Finally, the Seahorse conditions have been optimized for the 24-well format, so users will have to scale down the cells and reagents if the 96-well format is used.
2. High resolution LC-MS systems from other vendors (e.g., Thermo Exploris 240 Orbitrap, SCIEX triple TOF5600, Waters Xevo G2 QtoF, Shimazu LCMS-9030 QTOF) can also be used for the procedure.
3. MTBE is a volatile and colorless liquid, which is sparingly soluble in water. Studies have shown that MTBE can effectively replace chloroform for lipid extraction and deliver similar or better recoveries of all major lipid classes compared with the gold-standard Folch or Bligh and Dyer Recipes in which chloroform is used. The main advantage of MTBE extraction over conventional two-phase chloroform-containing solvent systems comes from the low density of the lipid-containing organic phase that forms the upper layer during phase separation. This simplifies the collection of the lipid phase and minimizes dripping losses. Furthermore, compared with chloroform, MTBE is nontoxic and noncarcinogenic.
4. Either chloroform/methanol or isopropanol/methanol can be used for the reconstitution of dried lipid extract. Isopropanol was selected because it is not as toxic as chloroform.
5. Pooled QC sample was injected periodically together with the samples to monitor the stability of the LCMS system. The coefficient of variance (CV) of each feature was calculated using the QC data and only the features with an CV < 20% were kept for the subsequent data analysis.
6. QTOF MS is a hybrid mass spectrometer that combines the benefits of two types of mass analyzer: quadrupole (good scan speed/sensitivity and robustness) and time-of-flight (high resolution and high mass accuracy). It offers three data acquisition modes: full scan, data-dependent acquisition, and data-independent acquisition, and is able to obtain high resolution precursor or product ion spectra. These features make QTOF MS an important tool for untargeted omics analysis.

## Recipes

1. **Substrate-limited media**
  - Prepare two tubes of substrate-limited media by adding the following to two tubes of Seahorse XF RPMI, pH 7.4:
    Add glutamine (200 mM) to a final concentration of 1 mM.
    Add FBS to a concentration of 1% (v/v).
  - To one tube, add a 1:1 mixture of BSA-conjugated linoleic acid and BSA-conjugated oleic acid to a final concentration of 2.5 mg/mL for each fatty acid.
  - To the second tube, add BSA-only to a final concentration of 5 mg/mL to serve as a control. (As there are two fatty acids added into the fatty acid–supplemented media, the BSA concentration in the BSA-only media should be 2× for the BSA concentration to be equivalent.)
  - Filter both tubes of substrate-limited media through a 0.22 μm filter and store at 4 °C.
2. **Seahorse assay media**
  - Prepare fatty acid–supplemented assay media by adding a 1:1 mixture of BSA-conjugated linoleic acid and BSA-conjugated oleic acid (2.5 mg/mL each) to Seahorse XF RPMI, pH 7.4
  - Prepare BSA-only assay media by adding 5 mg/mL of BSA to Seahorse XF RPMI, pH 7.4.
  - Store both sets of media 4 °C.
3. **10× drugs dilution**
  Dilute drugs stock (10,000×) to 10× on the same day as the Seahorse assay.
  - Oligomycin (10,000×,10 mM)
    Add 30 μL (10,000×) to 2,970 μL of Seahorse XF RPMI assay media.
  - FCCP (10,000×, 15 mM)
    Add 30 μL (10,000×) to 2,970 μL of Seahorse XF RPMI assay media.
  - Antimycin-A (10,000×, 10 mM) and rotenone (10,000×, 1 mM)
    Add 30 μL of rotenone (10,000×) and 30 μL of antimycin A (10,000×) to 2,940 μL of Seahorse XF RPMI assay media.
